# Books Reviewed

**Published:** 1863-05

**Authors:** 


### BOOKS REVIEWED.

*Clinical Lectures on the Diseases of Women and Children; By* GUNNING S. BEDFORD, A. M., M. D., *Professor of Obstetrics, the Diseases of Women and Children, and Clinical Obstetrics, in the University of New York; Author of the Principles and Practice of Obstetrics. Eighth edition, carefully revised and enlarged. New York:* WILLIAM WOOD & Co., 61, *Walker Street,* 1863.

This book has now passed to the eighth edition, and we have previously had occasion to make quite extended mention of its merits, but have always felt that the book far surpassed in excellence any compliments which its admirers could bestow. As our readers are all well aware, it has been translated into German and French, and has received the admiration and unqualified approbation of the profession, both in this country and in Europe. This last edition contains an additional chapter upon Carcinoma of the Uterus, and in this chapter we find some facts which, though perhaps not new, are yet so well stated and so full of interest that we propose giving some brief extracts, since it will be instructive, and at the same time give a truthful idea of the book we have under notice.

"While it cannot be said that cancer is a new malady, yet it may be affirmed that its true nature has been satisfactorily developed only within the last few years. For this development we are indebted to the researches of the Pathologist and the revelations of the Micographist. What was formerly regarded as carcinoma has been shown through these agencies to have been an entirely different affection. No longer will the medical man, if he move within the progress of scientific research, mistake, as was heretofore done, induration, engorgement, chronic inflammation, and other affections of the uterine organs for carmonmatous disease."

"Of all the organic lesions to which the uterus is liable, carcinoma is not only the most frequent, but beyond all comparison the most fatal.— Again, it has been shown by that able Pathologist, Rokitansky, that there is no organ in female economy which so often becomes the seat of this scourge of the sex as the uterus; and if, as it were, to make full the measures of woman's woes, next in the order of frequency are the mammæ."

"*Fatality of Cancer.*—Truly has it been remarked that death is the ordinary termination of cancer of the uterus; and we shall see, when discussing the treatment of this unrelenting malady, that the therapeutics of the practitioner will, unhappily, be more or less limited to the mitigation of symptoms as they develop themselves during the progress of this affection."

"*In what portion of the uterus does cancer usually commence and develop?* I think there is no fact more conclusively demonstrated than that the vaginal portion of the cervex of the organ is the ordinary seat of invasion."

"*At what age does cancer of the uterus most frequently occur?* There is a very general consent among authors that uterine cancer is a disease of advanced life, occuring most frequently between the fortieth and fiftieth years of age, or, in other words, about that climacteric of female existence, the cessation of the menstrual function."

"*Varieties of Cancer.*—There are four varieties of this disease. 1st—Scirrus; 2d—Medullary, or Encephaloid; 3d—Colloid, or Alveola; 4th—Epitheloid. I propose, very briefly, to examine some of the peculiarities of each of these forms of carcinoma." These varieties are very fully described, this section being very interesting and instructive, full and complete, and cannot be quoted in isolated portions, with fairness to the author; we therefore pass to the simple headings of the remaining portions of this lecture. "*Does cancer of the uterus involve abjacent structures?*" "*Duration of cancer of the uterus.*" "*The cause of cancer.*" *Symptoms: 1st, Hemorrhage; 2d, Pain; 3d, Vaginal Discharge; Constitutional changes.*

*Treatment.*—There are in the palliative management of carcinoma of the uterus three phenomena, which should especially attract the attention of the medical man, and call for his best efforts. They are as follows;—1st, Hemorrhage; 2d, Pain; 3d, The vaginal discharge. This chapter which has been added to the last edition of this book is a very valuable and important one, and fully completes the work. We have spoken particularly of this—not that it is more interesting than many others—but rather because it is a new one, and added to a book we have so often reviewed, so greatly admired, and heartily recommended.

---

*On Diseases of the Skin.* By Erasmus Wilson, F. R. S. *Fifth American from the fifth and revised London edition, with plates and illustrations on wood. Philadelphia:* Blanchard & Lea, 1863.

The high authority of this book upon the diseases of the skin is well known by all intelligent physicians. It has long been regarded as a text book of the greatest accuracy, containing full description of all the various diseases of the skin, with a nomenclature as faultless as possible, and an illustration more full and complete than elsewhere found.

This fifth edition comes to us greatly improved and illustrated by colored plates, representing the various skin diseases with unsurpassed accuracy. This mode of illustration is more important and perhaps more necessary in affections of the skin than in any other class of diseases, and with this improvement the book is really a great and almost indispensable addition to the library of every practitioner. The colored plates represent *urticaria, roseola, erythema, pemphigus, rupia, herpes, exzema, impetigo, ecthyma, lichen, strophulus, prurigo, lepra, pityriasis, psoriasis, lupus non exedens, acne, lycosis, trichosis, favus, exanthematous and papular syphilitic eruptions, tubercular syphilitic eruptions, diseases of the sebiparous organs*, and numerous wood illustrations. The value and importance of these representations of disease will become obvious to every physician, especially will physicians appreciate this who have not opportunity of seeing the diseases themselves in all their various appearances.

Skin disease is truly an important branch of general medicine, and demands for its thorough comprehension a most complete knowledge of general medicine, as well as particular and definite knowledge of the diseases which have their seat in the dermal textures. It requires careful observation of the phenomena presented, and thorough acquaintance with the symptoms, natural history, complications, causes and general appearance of skin disease, to be able to positively diagnose some of the most important of these maladies. This book by Erasmus Wilson is decidedly superior both in pictorial and typographic representation, and we most earnestly recommend it to the attention of our readers.

---

*A Theoretical and Practical Treatise on Midwifery, including the diseases of Pregnancy and parturition, and the attentions required by the Child from birth to the period of weaning.* By T. Cazeaux, *Member of the Imperial Academy of Medicines; Adjunct Professor in the faculty of Medicine of Paris; Chevelier of the Legion of Honor, and of the supplementary number of the order of Charles III; Member of the Surgical Society; of the Biological Society; of the Medical Society of Emulation; of the Anatomical Society; non-resident Associate of the Medical Society of Bordeaux; Correspondent of the Society of Ac-*

*coucheurs of Berlin; President of the Medical Society of the Department of the Seine. Adopted by the Superior Council of Instruction, and placed by Ministerial decision in the rank of classical works, designed for the use of Midwife Students in the Maternity Hospital of Paris—Third American, translated from the Sixth French edition.* By Wm. R. Bullock, M. D.; *with One Hundred and Forty Illustrations.* Philadelphia: Lindsay & Blackistone, 1863.

*Translator's Preface to the Third American edition.*—"In preparing for the press the *Third American* from the *Sixth* and last French edition of Cazeaux's Midwifery, the translator has endeavored carefully to make whatever alterations and corrections were required in the preceding editions. Many additions, which it would be difficult to point out in this place, have been made throughout the work, besides an article on the important subject of *Turning by External Manipulation.* The demand for a new American Edition is the best criterion of the estimation in which the work of Mr. Cazeaux is held in this country. The Translator hopes that it will be found to merit the increasing confidence of the profession.

Wm. R. Bullock."

*Wilmington, Delaware, Jan.* 23, 1863.

This work on Obstetrics is truly a very complete and valuable one, no topic in the whole range of Obstretical practice having escaped consideration; nothing has appeared too trivial for notice, and no one subject has been esteemed so all-important as to exclude other and equally necessary matter for a complete treatise upon Obstretic Medicine.

The high reputation of the author and his unlimited opportunity of observation entitle his opinions to great respect, and the profession of this country will not be able to find any work translated from the French on Obstretics, or indeed from any language, more fully entitled to considerations and respect than Cazeaux's Midwifery. The illustrations are worthy also of especial mention. The plate representing the fœtal circulation is most perfect and beautiful, itself worth more than the cost of the book. The Publishers have done themselves, as well as the work, great honor, by the substantial and finished style in which it is presented to the profession.

Considering it from all points of view, it is one of our best standard works upon Obstetrics and diseases of Pregnancy, containing full instruction upon all subjects within the scope of its design, and most fully entitled to its present name and fame.

We cannot speak very fully upon the point of improvement in this last American edition since we have not the former, but have read the remarks in the article on "Turning by External Manipulation" and have been greatly interested in the discussion. The description of the process, proper time, and circumstances, and favorable results, are all worthy of consideration and should be understood by practitioners. There are other numerous additions and improvements, and we have no doubt that this edition of the book will be received with even greater favor than the preceding ones, by the profession.

---

